# Testing for HIV/STIs in China: Challenges, Opportunities, and Innovations

**DOI:** 10.1155/2017/2545840

**Published:** 2017-02-19

**Authors:** Huachun Zou, Lei Zhang, Eric P. F. Chow, Weiming Tang, Zixin Wang

**Affiliations:** ^1^Department of Medical Statistics and Epidemiology, School of Public Health, Sun Yat-sen University, Guangzhou 510080, China; ^2^Kirby Institute, The University of New South Wales, Sydney, NSW 2052, Australia; ^3^Melbourne Sexual Health Centre, Alfred Health, Melbourne, VIC 3053, Australia; ^4^Central Clinical School, Monash University, Melbourne, VIC 3004, Australia; ^5^School of Public Health and Preventive Medicine, Faculty of Medicine, Monash University, Melbourne, VIC 3004, Australia; ^6^Research Centre for Public Health, Tsinghua University, Beijing 100084, China; ^7^University of North Carolina Project-China, Guangzhou 510085, China; ^8^JC School of Public Health and Primary Care, Faculty of Medicine, The Chinese University of Hong Kong, Shatin, Hong Kong; ^9^Shenzhen Research Institute, The Chinese University of Hong Kong, Shenzhen 518057, China

In this special issue, five articles are included. The research topics include the trend of late presentation in HIV-infected populations in Guangzhou, China, positivity of human papillomavirus (HPV) among HIV-infected and HIV-uninfected men who have sex with men (MSM) in Guangzhou, China, association between age at first anal/vaginal sex and HIV infection among MSM in Shenzhen, China, impacts of online sex-seeking on HIV transmission among MSM in Shenyang, China, and the prevalence of antiretroviral drug resistance in people living with HIV in Jiangsu, China. Factors that are associated with HIV infection and challenges encountered by people living with HIV in different geographical areas of China are discussed, with MSM being the focus. These articles will hopefully provide evidence and insights to policy makers for HIV/STI control and prevention in China.

Using data extracted from the Chinese HIV/AIDS Comprehensive Response Information Management System (CRIMS), W. Cheng et al. examined the prevalence and trend of late presentation of HIV infection and explored the roles of different testing strategies [including voluntary counseling and testing (VCT) centers, provider-initiated testing and counseling (PITC) services at general hospitals and clinics, and Mandatory HIV Testing (MHT) at detention facilities] in HIV diagnosis in Guangzhou, China. The results showed that, in metropolitan city of Guangzhou, late presentation of HIV infection, defined as CD4 cell count below 350 cells/*μ*l, decreased from 63% in 2008 to 43% in 2013. Likewise, presentation with an advanced HIV disease decreased from 40% in 2008 to 15% in 2013. Despite these reductions, late presentation of HIV infection at diagnosis remained high. The results revealed that, compared to PITC and MHT, VCT was a more efficient platform in early HIV diagnosis. PITC did not facilitate more timely diagnosis, which highlighted the gap of intervention to raise HIV awareness among key populations attending general health care facilities. The results also suggested that MHT allowed healthcare providers to provide early intervention to the key populations and to provide opportunities for testing, substance abuse treatment, and antiretroviral treatment (ART), which played an important role in the control of HIV epidemic. Recently, the updated national ART guideline recommends free ART for all PLWH upon diagnosis. Early identification of HIV-infected cases facilitates early treatment, which can in turn reduce the chance of onward HIV transmission.

X. Ren et al. reported on the positivity of anal HPV among HIV-positive and HIV-negative men who have sex with men (MSM) attending a sexual health clinic based at a gay community organization in Guangzhou. This study was timely to add to the knowledge of the epidemic of HPV among MSM in China. The sample was predominantly young (median 26 years) and sexually experienced (40% had two or more partners in the past three months) MSM. Positivity of HPV of any type, any high-risk type, any low-risk type, any 4-valent vaccine type, and any 9-valent vaccine type in HIV-infected MSM, was almost twice as high as that in their HIV-uninfected counterparts. This study also found that prevalent anal bacterial infections, including chlamydia, gonorrhea, and* Mycoplasma genitalium*, contributed to much higher anal HPV positivity and multiplicity of anal HPV types, which implied the importance of timely detection and treatment of bacterial infections. The vast majority of HPV-infected MSM were infected with 9-valent vaccine types (59 out of 64 HIV-infected ones and 31 out of 41 HIV-uninfected ones). The high positivities of high-risk types including HPV 16, 18, and 52 and low-risk types including HPV 6 and 11 in the anal canal in MSM, especially in those infected with HIV, suggested high burdens of corresponding morbidities in the foreseeable future, including anal warts that may relapse, anal intraepithelial neoplasia, and even anal cancer. The study results warranted early HPV vaccination and regular anal exams in MSM attending sexual health clinics in Guangzhou. In 2016 the health authority in China approved the bivalent HPV vaccine manufactured by GlaxoSmithKline to be used in women. However HPV vaccination in males is rarely discussed. The acceptability of HPV vaccination in Chinese MSM is high, especially when it is provided free of charge. A study in Hong Kong, a city close to Guangzhou, found that over 80% of MSM were willing to take up free HPV vaccines [1]. However, the unavailability of policies with regard to HPV vaccination in men and targeted HPV vaccination in MSM in China remains to be an issue for the advocacy of policy changes.

R. Xu et al. reported the association between age at first anal sex with a man or vaginal sex with a woman and HIV infection among 533 MSM attending a sexual health clinic in Shenzhen. This sample was relatively young (median 32 years), commenced sex relatively late (21 years for vaginal sex with a woman and 22 years for anal sex with a man), and had a high rate of HIV infection (24%). A large proportion (66%) had ever had sex with women. MSM who also have sex with women is a bridge population transmitting HIV/STIs from high-risk population to low-risk population (e.g., female sex partners). Being younger, being unmarried/divorced/widowed, having lower education level, and being socioeconomically disconnected were associated with a relatively younger age at first sexual intercourse. Commencing anal sex before 14 was associated with higher rate of HIV infection. The proportion of consistent condom use in MSM in China is still suboptimal. This study called for the promotion of consistent condom use in anal sex and early education and interventions to be conducted among MSM. But given the availability of alternative biomedical and behavioral interventions for HIV, including preexposure prophylaxis and serosorting, it is quite difficult to increase condom use.

S. Pan et al. conducted a 6-year serial cross-sectional survey to study on the impacts of online sex-seeking (including using smartphone-based geosocial networking apps) and HIV infection among MSM in Shenyang, China. Around half of the participants (1000/1981) sought sex partners mainly through the Internet (Internet-based MSM, IBM) and this rate increased from 43% in 2009 to 62% in 2014. This study confirmed results from a meta-analysis on global data: IBM were younger, had a higher level of education, and had higher income, compared to non-IBM [2]. This study found a changing trend in HIV prevalence among IBM compared to non-IBM. Before 2010, the prevalence of HIV among IBM was slightly lower than non-IBM. But from 2010 to 2014, HIV prevalence in IBM increased from 12.5% to 20.7%, while that in non-IBM was stable (from 13.5% to 14.7%). Since May 2009, MSM have been increasingly using mobile apps to socialize in the gay community [2]. Sex-seeking using smartphone-based geosocial networking apps may have contributed to the dramatic increase of number of partners and unprotected anal sex. IBM preferred receptive anal sex and had a higher rate of STD symptoms. MSM engaging in receptive anal sex were 7 times more likely to be infected with HIV compared to MSM engaging in insertive anal sex [3]. All these factors contributed to a higher HIV prevalence among IBM. The study results can help public health policy makers to understand the HIV transmission routes among MSM and provide effective and comprehensive interventions. Given that most MSM are now using apps, interventions can potentially be done via apps.

Y. Zhou et al. analyzed the prevalence of ART drug resistance and its impact on HIV-1 virological failures in Jiangsu, China. A total of 2223 HIV-infected patients who had received ART for at least one year were included. Just over 10% had a viral load of >1,000 copies/mL (i.e., virologic failure), half of whom had drug resistance to either nucleoside reverse transcriptase inhibitors (NRTIs), nucleoside reverse transcriptase inhibitors (NNRTIs), or protease inhibitors (PIs). The overall prevalence of drug resistance was 5% (101/2223). Among these patients, the highest frequency drug resistance mutations were NNRTIs (47%), followed by NRTIs (37%), and PIs (6%). The most common mutations associated with drug resistance in NRTIs and NNRTIs were M184V (79%) and K103N (34%). Four PI-associated mutations were observed in 11 individuals. This study called for the scale-up of testing for transmitted drug resistance (TDR) in treatment-naive HIV-1 infected patients, to better monitor the epidemic. MSM tended to have higher drug resistance rates and worse treatment outcomes compared to other populations, and thus additional monitoring and further assessments of the drug resistance profile in this population are necessary.

*Huachun Zou* *Lei Zhang* *Eric P. F. Chow* *Weiming Tang* *Zixin Wang*

## References

[1] Lau J. T. F., Wang Z., Lau M., Lai C. H. Y. (2014). Perceptions of HPV, genital warts, and penile/anal cancer and high-risk sexual behaviors among men who have sex with men in Hong Kong. *Archives of Sexual Behavior*.

[2] Zou H., Fan S. (2016). Characteristics of men who have sex with men who use smartphone geosocial networking applications and implications for HIV interventions: a systematic review and meta-analysis. *Archives of Sexual Behavior*.

[3] Meng X., Zou H., Fan S. (2015). Relative risk for HIV infection among men who have sex with men engaging in different roles in anal sex: a systematic review and meta-analysis on global data. *AIDS and Behavior*.

